# Antinociceptive Effect and HPLC Profile of Lyophilized Chicory and Oregano Decoction

**DOI:** 10.3390/plants15040527

**Published:** 2026-02-08

**Authors:** Ivana Zlatanović Đaić, Ivana Dimitrijević, Sonja Ilić, Katarina Mitić Ivković, Nenad Stojiljković, Gordana Stojanović

**Affiliations:** 1 Department of Chemistry, Faculty of Sciences and Mathematics, University of Niš, Višegradska 33, 18000 Niš, Serbia; ivana.zrnzevic@pmf.edu.rs (I.D.); gordana.stojanovic@pmf.edu.rs (G.S.); 2 Department of Physiology, Faculty of Medicine, University of Niš, Bulevar Dr. Zoran Đinđić 81, 18000 Niš, Serbia; sonja.ilic@medfak.ni.ac.rs (S.I.); nenad.stojiljkovic@medfak.ni.ac.rs (N.S.)

**Keywords:** oregano, chicory, lyophilized decoction, HPLC-DAD analysis, antinociceptive potential

## Abstract

The chemical composition and antinociceptive potential of a lyophilized decoction of the oregano flowers (*Origanum vulgare* L.) and the aerial parts of chicory (*Cichorium intybus* L.) in the flowering phase (LCOD—lyophilized decoction of the oregano and chicory) was investigated by HPLC-DAD and the acetic-acid-induced writhing method. HPLC-DAD analysis of the LCOD revealed the presence of 20 phenolic compounds, where the dominant phenolic components were ferulic acid (205.19 mg/g LCOD), rosmarinic acid (81.55 mg/g) and hyperoside (79.42 mg/g). The results of the antinociceptive activity showed a strong analgesic effect of the LCOD (15 and 30 mg/kg), which significantly (*p* < 0.001) reduced the number of writhings (98.40 and 99.23%, respectively) induced by acetic acid. These encouraging results indicate the analgesic potential of LCOD and suggest validation through clinical trials.

## 1. Introduction

Primary dysmenorrhea is a painful condition in a woman that recurs every month during the reproductive period. Non-steroidal anti-inflammatory drugs are often used to relieve menstrual symptoms (abdominal cramps, diarrhea, constipation, stomach pain and heavy bleeding) [1,2]. In overcoming the excessive use of analgesic agents, the use of available and cheap herbal preparations could be helpful.

A tea obtained from chicory and oregano is traditionally used for menstrual pain in the rural areas of the Nišava district. Numerous pharmacological effects and medicinal uses of oregano and chicory have been described. Oregano, as a flavoring herb, is used in traditional medicine due to its health effects, mainly for the treatment of colds, indigestion and stomach complaints, for bronchial diseases and as a digestive and antiseptic agent. Numerous studies have documented its anticancer, anti-inflammatory, antioxidant and antimicrobial activities [3]. Chicory has been recognized since antiquity for its medicinal properties and uses for strengthening the prostate and other reproductive organs and diarrhea, lung diseases and coughs, cancer, liver problems, stomach cramps, hemorrhoids, rashes, etc. It exhibits multifaceted biological activities, including hepatoprotective [4], anti-inflammatory [5], antioxidant [6], antidiabetic, hypolipidemic [7], antimicrobial, anti-protozoal [8], analgesic, sedative [9], gastroprotective, cardiovascular, anticancer, reproductive, immunological, anthelmintic, wound healing and many other pharmacological effects [10]. Chicory extracts obtained from leaves with 1% hydrochloric acid were tested for the inhibition of cyclooxygenase (COX-1 and COX-2) and lipid peroxidation (LPO) activity. The results confirmed that the treatment inhibited LPO activity in the range of 54.5–92%, COX-1 (a constitutive isoform of cyclooxygenase) activity in the range of 15.6–41.3% and COX-2 (induced cyclooxygenase) activity in the range of 43.7–84.9%, compared with the control samples [11]. The action on COX-1 and COX-2 enzymes is important because they catalyze the conversion of arachidonic acid into prostaglandins, which play a key role in inflammation and pain perception. Considering the use of a tea mixture of oregano and chicory in traditional medicine for pain relief and its proven effect on inhibiting enzymes that are responsible for the sensation of pain, we assumed that the mixture of oregano and chicory would exhibit analgesic effects.

Recent studies confirm that rosmarinic acid remains the predominant phenolic constituent of oregano, which is particularly concentrated in the floral parts. This is accompanied by significant amounts of salvianolic acid and caffeic acid derivatives. The flavonoid fraction of oregano is characterized by the presence of flavones, such as luteolin and apigenin, along with their respective glycosides [12,13,14]. The aerial parts of chicory are recognized as a primary source of hydroxycinnamic acid derivatives, with chicoric acid, caftaric acid, and chlorogenic acid being the most prominent markers. Recent investigations suggest that ferulic acid and its derivatives may contribute to the plant’s pharmacological efficacy, including the modulation of prostaglandin-mediated pathways. The polyphenolic profile of the aerial parts is further enriched by flavonol glycosides: predominantly hyperoside and rutin [15,16,17].

To the best of our knowledge, there are no published papers on the chemical composition of the chicory and oregano lyophilized decoction (LCOD) or on its analgesic activity on mice.

The selection of *Origanum vulgare* L. flowers and the aerial parts of *Cichorium intybus* L. for this study was primarily based on compelling ethnopharmacological evidence from the Nišava District (Southeastern Serbia). In this region, a decoction prepared from this specific herbal mixture has a long-standing tradition in folk medicine for alleviating visceral pain, particularly symptoms associated with dysmenorrhea. The initial hypothesis for this research was further supported by empirical observations and positive anecdotal reports within the authors’ local environment, where the efficacy of this traditional preparation has been consistently noted. Despite its widespread use, these traditional claims had not been previously validated through rigorous phytochemical and pharmacological testing. Therefore, this study was designed to evaluate the scientific basis for the use of this traditional preparation, effectively bridging the gap between regional folk knowledge and evidence-based phytotherapy.

The primary objective of this work was to investigate the detailed chemical composition and antinociceptive potential of a lyophilized chicory and oregano decoction (LCOD). The use of the lyophilized form (powder) offers significant practical advantages over traditional liquid decoctions, as it ensures standardized dosing, ease of preparation, and enhanced stability. By focusing on the combined decoction, this study aims to evaluate the potency of the herbal mixture exactly as it is utilized in ethnopharmacological practice, providing a foundation for its potential development as a natural and cost-effective alternative to synthetic analgesics.

## 2. Results and Discussion

### 2.1. HPLC Analysis

HPLC-DAD analysis of the lyophilized chicory and oregano decoction (LCOD) revealed a complex profile consisting of ten phenolic acids and ten flavonoids (Table 1). The phenolic acid fraction included caftaric acid and its derivative, protocatechuic, chicoric (and its derivative), lithospermic, and rosmarinic acids, alongside caffeic and ferulic acid derivatives. The flavonoid profile was characterized by the presence of catechin, epigallocatechin, epicatechin, hyperoside, kaempferol-3-O-glucoside, rutin, luteolin, and derivatives of kaempferol-glycoside, apigenin-C-hexoside, and quercetin.

**Figure 1:**
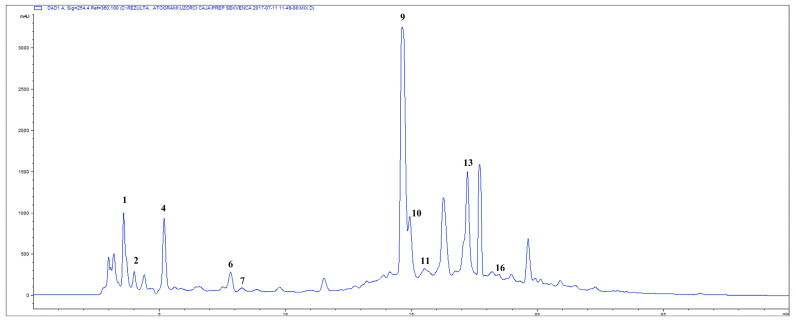
HPLC–DAD chromatogram of LCOD recorded at 254 nm. Numbered peaks correspond to the compounds identified and listed in Table 1.

**Figure 2:**
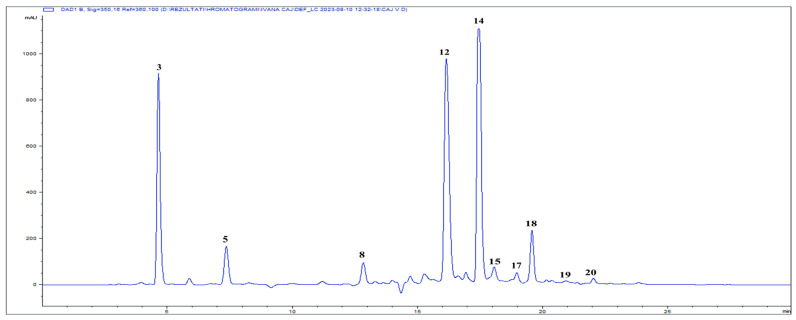
HPLC–DAD chromatogram of LCOD recorded at 350 nm. Numbered peaks correspond to the compounds identified and listed in Table 1.

Phytochemical constituents were identified and quantified using HPLC chromatograms recorded at 254 and 350 nm (Figure 1 and Figure 2, and Supplementary Materials). These wavelengths were selected based on the UV-Vis absorption maxima of the target analytes to maximize detection sensitivity. Ferulic acid emerged as the most abundant constituent (205.19 mg/g), followed by rosmarinic acid (81.55 mg/g), hyperoside (79.42 mg/g), and caftaric acid (60.96 mg/g).

The chemical signature of the decoction represents a clear hybrid of its parental botanical sources. Rosmarinic and lithospermic acids (49.85 mg/g) originate from the oregano flowers (*Origanum vulgare* L.), while the high levels of caftaric, chicoric (29.55 mg/g), and ferulic acids are characteristic markers of the chicory aerial parts (*Cichorium intybus* L.). The presence of ferulic acid in *O. vulgare* has been previously documented [18,19], while recent studies have also confirmed its occurrence in aqueous extracts of *C. intybus* [15].

Furthermore, the discovery of protocatechuic acid in LCOD is consistent with reports on *O. vulgare* analyzed via LC-MS/MS [19]. Notably, Martins et al. [20] identified protocatechuic acid in *O. vulgare* decoctions and infusions, albeit at a lower concentration (~1 mg/g) compared to the levels found in LCOD. Similarly, Aljumayi [21] identified protocatechuic acid as a primary phenolic acid in chicory extracts.

Among the flavonoids, hyperoside showed the highest concentration (79.42 mg/g), followed by epigallocatechin (23.86 mg/g), kaempferol-3-O-glucoside (19.68 mg/g), epicatechin (15.41 mg/g), apigenin-C-hexoside (14.76 mg/g), catechin (12.42 mg/g), and rutin (9.96 mg/g). Minor constituents included luteolin, quercetin derivative, and kaempferol-glycoside derivative (1.44–5.41 mg/g). These findings align with previous studies where these flavonoids were detected in *O. vulgare* [19,22,23,24] and *C. intybus* [25,26], using various extraction solvents.

### 2.2. Acetic Acid-Induced Writhing Method

The acetic acid-induced writhing model was selected as a well-established paradigm for visceral pain. Its underlying mechanism involves the release of inflammatory mediators and prostaglandins in the peritoneal cavity, closely mimicking the pathophysiology of dysmenorrhea.

The results (Table 2) demonstrate that LCOD possesses extraordinary antinociceptive potency, which correlates directly with its complex polyphenolic profile. A ceiling effect was observed at a dose of 50 mg/kg, with no significant difference in inhibition between the 15 mg/kg (98.40%) and 30 mg/kg (99.23%) doses. Notably, LCOD exhibited significantly higher efficacy than pure rosmarinic acid; while rosmarinic acid (100 mg/kg) achieved 67.73% inhibition, LCOD at 15 mg/kg yielded near-total inhibition (98.40%).

This suggests a profound synergistic entourage effect, where minor and major compounds enhance each other’s bioavailability or pharmacological action. Ferulic acid, the most abundant component (205.19 mg/g), is well-documented for its ability to suppress pro-inflammatory mediators. Our findings complement those of Kaşık et al. [27], who reported that ferulic acid (80 mg/kg) significantly inhibited abdominal contractions (65.89%), comparable to acetylsalicylic acid. Similarly, the results for rosmarinic acid (50 and 100 mg/kg) in our study align with those of Boonyarikpunchai et al. [28], who observed 52% and 85% inhibition, respectively. Protocatechuic acid also contributes to this profile, as it is known to produce dose-dependent analgesic effects [29].

The high concentration of these phenolic acids likely plays a pivotal role in desensitizing peripheral nociceptors. The synergistic framework of ferulic, rosmarinic, and chicoric acids—known inhibitors of the COX and LOX pathways—explains why LCOD at 30 mg/kg outperformed the clinical standard, diclofenac sodium (80.65%). Additionally, flavonoids like hyperoside and rutin provide another layer of protection by modulating protein kinases and inhibiting bradykinin release. The presence of luteolin and apigenin derivatives may further suggest a potential central mechanism of action.

In summary, the superior antinociceptive activity of LCOD is a consequence of the rich concentration of hydroxycinnamic acids and a diverse array of flavonoids. This phytochemical synergy enables LCOD to achieve maximal analgesic effects at exceptionally low doses, positioning it as a potent natural alternative to conventional NSAIDs.

Although a comparative evaluation of the individual plant extracts was beyond the scope of this study due to ethical considerations regarding animal welfare, preliminary profiling suggests that the co-boiling process may facilitate unique phytochemical interactions within the LCOD matrix. Elucidating these molecular mechanisms remains a research priority. Given that chicory and oregano are widely distributed and accessible, this traditional decoction represents a highly cost-effective and efficacious strategy for the management of visceral pain.

While this study focused on the 1:1 ratio used in traditional medicine to validate its local application, future research should explore different component ratios to optimize the synergistic potential of the blend.

To the best of our knowledge, there are no published papers on the chemical com-position of the LCOD as well as on its analgesic action in mice and the possible mechanism of its action.

## 3. Materials and Methods

### 3.1. Plant Material

*Origanum vulgare* L. was collected during the flowering period from June to August, on mountain Seličevica, Serbia (coordinates 43°14′10″ N; 21°58′12″ E). *Cichorium intybus* L. (known as blue daisy and coffeeweed) was collected during July in the village Brestovac, Serbia (43°09′14″ N; 21°52′28″ E coordinates). The voucher specimens were deposited in the “Herbarium Moesiacum Niš” (HMN) of the Department of Biology and Ecology, Faculty of Sciences and Mathematics, University of Niš, under the acquisition numbers 14,610-F and 14,602, respectively. Plant identification was carried out by Dr. Bojan Zlatković, a full professor at the Department of Biology and Ecology, Faculty of Sciences and Mathematics, University of Niš.

### 3.2. Extraction

The decoction was made from 6 g of dried oregano flowers and 6 g of chopped above-ground part of dried chicory with 200 mL of cold water by heating to boiling. Afterwards, the decoction was concentrated to a small volume on a rotary vacuum evaporator (KNF Laboxact, Freiburg, Germany) (45 °C) and lyophilized by Alpha 1–2 LDplus freeze-dryer (CHRIST, Osterode, Germany) working in automatic mode, equipped with an oil vacuum pump (max 4 × 10^−4^ mbar). The concentrated decoction was frozen at −30 °C in a freezer chamber and further freeze-dried for 15 h at a temperature ranging from −20 °C to −26 °C and in a vacuum from 1 mbar to 0.2 mbar (in main drying mode); the final drying duration was 8 h, at a temperature that ranged from −35 °C to −38 °C and a vacuum from 0.07 to 0.06 mbar. The working temperature of the ice condenser was −55 °C. The amount of powder lyophilisate obtained was 2.7942 g.

### 3.3. HPLC Analysis

HPLC analysis was performed on an Agilent Santa Clara, CA, USA), Zorbax Eclipse XDB-C18, 5 μm, 4.6 mm × 150 mm column (Agilent Technologies, Santa Clara, CA, USA), using an Agilent 1200 series liquid chromatograph (equipped with a diode array detector (DAD), Chemstation Software, a quaternary pump, an online vacuum degasser, auto sampler and a thermostated column compartment. Data were acquired and processed using Agilent ChemStation software, version B.04.03 (Agilent Technologies, Santa Clara, CA, USA). The mobile phase consisted of two solvents: water–formic acid (1%) (A) and methanol (B) (HPLC grade). The elution program was as follows: from 0 to 5 min, 30% B was used; from 5 to 20 min, the proportion of solvent B was gradually increased to 70%; from 20 to 25 min, it was further gradually increased to 90% B; and from 25 to 40 min, 90% B was maintained. The flow rate was 0.5 mL/min and the injection volume was 20 μL of solution, obtained by dissolving 10 mg of lyophilisate in 2 mL of deionized water. The column was thermostated at 25 °C. Data from all peaks were accumulated in the range 190–400 nm, and chromatograms were recorded at 254, 280, 350 and 520 nm. Identification of compounds was conducted by comparing the retention time and UV spectra with those obtained from the available standard solution. Otherwise, peaks were tentatively identified by comparing the obtained data with the available spectra in the literature. Quantification was performed using calibration curves for gallic acid (Sigma-Aldrich, St. Louis, MO, USA), chicoric acid (Sigma-Aldrich), ferulic acid (Sigma-Aldrich), rosmarinic acid (Sigma-Aldrich), lithospermic acid (Phytolab GMBH & CO. KG, Vestenhoff, Germany), rutin (Sigma-Aldrich), quercetin (Sigma-Aldrich) and catechin (Sigma-Aldrich). All analytical standards used for quantification were of a high purity (≥94–98%, HPLC grade). The amount of identified compounds was expressed as mg per g of lyophilisate. All standards were supplied by Sigma-Aldrich except lithospermic acid, which was purchased from Phytolab GMBH & CO. KG.

Due to the unavailability of commercial standards for all identified constituents, quantification was performed using surrogate standards with similar chemical structures. Specifically, rutin was employed, as is standard for hyperoside, and kaempferol and apigenin derivatives; gallic acid for protocatechuic acid; quercetin for quercetin derivatives and luteolin; and rosmarinic acid was used for caftaric acid and its derivative.

### 3.4. Acetic Acid-Induced Writhing Method

The animals used in the experiment were maintained under standard laboratory conditions (22 ± 2 °C, 60% humidity, and a 12 h light/dark cycle), with access to food and water ad libitum. All experimental procedures were conducted in accordance with the ethical guidelines of the European Union (Directive 2010/63/EU) and the regulations of the Republic of Serbia (No. 323-07-03858/2023-05).

In this study, 42 Balb/c mice (an albino) were used, weighing an average of 25 g, divided into 7 groups of 6 animals each. Group AA was treated by intraperitoneal (i.p.) injection with 1.2% acetic acid (10 mL/kg) (Sigma-Aldrich, St. Louis, MO, USA; 99.7% purity). D group received i.p. commercial analgesic diclofenac sodium (Hemofarm A.D., Vršac, Republic of Serbia; 99% purity) in a dose of 10 mg/kg, 30 min before the application of acetic acid. Lyophilized chicory and oregano decoction (LCOD) at doses of 5 mg/kg (T5 group), 15 mg/kg (T15 group) and 30 mg/kg (T30 group) and rosmarinic acid at doses of 50 mg/kg (RA50 group) and 100 mg/kg (RA100 group) were administered orally, 30 min before acetic acid administration. The number of writhings was registered in the period from the fifth to the twentieth minute from the moment of application of acetic acid. Antinociceptive activity was expressed as inhibition of writhing in percent, using the following formula:

(1) $$\%\;\mathrm{inhibition}=\frac{C-T}{C}\times 100,$$

where *C* is the average number of writhings in the group treated with acetic acid, and *T* is the average number of writhings in the test group.

The doses of 50 and 100 mg/kg for pure rosmarinic acid were strictly based on the established literature data concerning its known analgesic activity in rodent models, ensuring comparability with existing research. The doses for the lyophilized decoction (LCOD) (5, 15, and 30 mg/kg) were determined following a preliminary dose-finding study. An initial high dose of 50 mg/kg was tested, which resulted in complete inhibition of the writhing response (100% inhibition), indicating a ceiling effect. Consequently, a series of lower, logarithmic doses (5, 15, and 30 mg/kg) were selected to accurately establish the dose–response curve and determine the minimum effective dose (ED50), which was a primary goal of our investigation.

Parameter values are expressed as mean ± standard deviation. Statistical significance was determined by one-way analysis of variance (ANOVA) and post hoc Tukey’s test. A value of *p* < 0.05 was considered to be statistically significant. Statistical analysis of the results was performed using the Graphpad Prism 5.03 computer program, San Diego, CA, USA.

## 4. Conclusions

This study characterized the polyphenolic profile of a lyophilized decoction prepared from a mixture of oregano and chicory (LCOD) and evaluated its antinociceptive potential in a mouse model of acute visceral pain. HPLC-DAD profiling identified ferulic acid, rosmarinic acid, and hyperoside as the predominant constituents of the LCOD matrix. These compounds are well-recognized for their diverse pharmacological properties, particularly their potent antioxidant and anti-inflammatory activities, which underpin the traditional use of these plants in various healing systems worldwide.

Our pharmacological results demonstrate that LCOD possesses an exceptional analgesic effect, outperforming the clinical standard, diclofenac sodium. Specifically, LCOD at doses of 15 and 30 mg/kg achieved near-total inhibition of the writhing response (98.40% and 99.23%, respectively), compared to the 80.65% inhibition observed for Diclofenac. The finding that LCOD exhibits significantly higher potency than pure rosmarinic acid suggests a profound synergistic interaction between the decoction’s constituents. This indicates that the overall activity is a result of complex interplay, likely dominated by the ferulic–rosmarinic acid axis, which warrants further investigation through multi-component dose–response studies.

From a practical perspective, the lyophilized form of the decoction offers a superior alternative to traditional preparation methods. Unlike the time-consuming process of boiling, straining, and cooling large volumes of liquid, the lyophilized powder allows for rapid dissolution, standardized dosing, and enhanced stability. This makes LCOD a highly effective, practical, and cost-efficient natural candidate for the management of visceral pain, providing a scientific basis for its traditional application in alleviating symptoms such as dysmenorrhea.

## Figures and Tables

**Table 1:** Retention time, UV–Vis absorption maxima, compound classes, and quantitative composition (mean ± SD, mg/g) of phenolic compounds identified in the lyophilized decoction of *O. vulgare* and *C. intybus* (LCOD) by HPLC–DAD.

N	Rt	λ_max_	Class	Compound	LCOD (mg/g) ± SD
1	3.58	230, 252, 293	F	Epigallocatechin	23.86 ± 0.95
2	3.99	228, 255, 290	F	Catechin	12.42 ± 0.50
3	4.64	235, 290, 343	A	Caftaric acid *	60.96 ± 1.80
4	5.18	227, 260, 298	A	Protocatechuic acid *	7.78 ± 0.31
5	7.35	234, 295, 343	A	Caftaric acid derivative *	13.55 ± 0.54
6	7.82	226, 255	F	Epicatechin	15.41 ± 0.62
7	8.26	230, 255, 295, 350	A	Caffeic acid derivative *	7.66 ± 0.30
8	12.83	238, 298, 332	A	Chicoric acid	29.55 ± 1.18
9	14.62	230, 263, 297	A	Ferulic acid	205.19 ± 4.10
10	14.92	232, 263, 293	A	Ferulic acid derivative *	10.24 ± 0.41
11	15.51	234, 289, 329	A	Chicoric acid derivative *	11.60 ± 0.46
12	16.14	255, 267, 347	F	Hyperoside *	79.42 ± 2.38
13	17.21	235, 255, 285, 310	A	Lithospermic acid	49.85 ± 1.99
14	17.44	234, 287, 328	A	Rosmarinic acid	81.55 ± 2.45
15	18.06	238, 265, 341	F	Apigenin-C-hexoside *	14.76 ± 0.59
16	18.45	225, 260, 340	F	Kaempferol-glycoside derivative *	1.44 ± 0.11
17	18.96	225, 255, 360	F	Rutin	9.96 ± 0.40
18	19.57	230, 256, 348	F	Kaempferol-3-O-glucoside *	19.68 ± 0.79
19	20.92	242, 334	F	Quercetin derivative *	3.10 ± 0.19
20	22.02	227, 254, 348	F	Luteolin *	5.41 ± 0.27
				Total	663.39

N—Peak number in the chromatogram, Rt—retention time (min), λ_max_—UV-Vis absorption maxima (nm), A—phenolic acid compounds, and F—flavonoid compounds. Compounds were identified by retention times and UV spectra compared with the standards and the literature. * Compounds quantified using the calibration curves of structurally related (surrogate) standards: gallic acid (for protocatechuic acid), rosmarinic acid (for caftaric acid and its derivative), ferulic acid (for ferulic acid derivative and caffeic acid derivative), chicoric acid (for chicoric acid derivative), rutin (hyperoside, and kaempferol and apigenin derivatives) and quercetin (for quercetin derivative and luteolin).

**Table 2:** Antinociceptive effect of lyophilized chicory and oregano decoction (LCOD), and rosmarinic acid in the acetic acid-induced pain model.

Treatment	Number of Writhings	Inhibition (%)
Acetic acid (10 mL/kg)	20.67 ± 1.75	-
Diclofenac sodium (10 mg/kg)	4.00 ± 0.89	80.65
LCOD 5 (5 mg/kg)	6.33 ± 2.42	69.37
LCOD 15 (15 mg/kg)	0.33 ± 0.51	98.40 *
LCOD 30 (30 mg/kg)	0.16 ± 0.41	99.23 *
Rosmarinic acid 50 (50 mg/kg)	10.67 ± 1.03	48.38
Rosmarinic acid 100 (100 mg/kg)	6.67 ± 0.82	67.73

* Statistically significant differences between the obtained results for the significance level *p* < 0.001.

## Data Availability

The authors confirm that the data supporting the findings of this study are available within the article.

## Supplementary Materials

The following supporting information can be downloaded at: https://www.mdpi.com/article/10.3390/plants15040527/s1, Figure S1: HPLC–DAD chromatogram of galic acid recorded at 254 nm; Figure S2: HPLC–DAD chromatogram of chicoric acid recorded at 350 nm; Figure S3: HPLC–DAD chromatogram of trans-ferulic acid recorded at 254 nm; Figure S4: HPLC–DAD chromatogram of lithospermic acid recorded at 350 nm; Figure S5: HPLC–DAD chromatogram of rutin recorded at 350 nm; Figure S6: HPLC–DAD chromatogram of rosmarinic acid recorded at 350 nm; Figure S7: HPLC–DAD chromatogram of quercetin recorded at 350 nm; Figure S8: UV-Vis spectrum of caftaric acid derivative. Figure S9: UV-Vis spectrum of chicoric acid derivative; Figure S10: UV-Vis spectrum of caffeic acid derivative; Figure S11: UV-Vis spectrum of ferulic acid derivative; Figure S12: UV-Vis spectrum of apigenin-C-hexoside; Figure S13: UV-Vis spectrum of kaempferol-3-O-glucoside; Figure S14: UV-Vis spectrum of kaempferol-glycoside derivative; Figure S15: UV-Vis spectrum of quercetin derivatives; Figure S16: UV-Vis spectrum of gallic acid; Figure S17: UV-Vis spectrum of caftaric acid; Figure S18: UV-Vis spectrum of chicoric acid; Figure S19: UV-Vis spectrum of ferulic acid; Figure S20: UV-Vis spectrum of quercetin; Table S1: Regression equations, correlation coefficients, retention times and linear ranges for the reference standards used for HPLC quantification.

## Abbreviation

The following abbreviation is used in this manuscript:

LCOD	Lyophilized decoction of the oregano and chicory
