# Supplementary material for: Antinociceptive Effect and HPLC Profile of Lyophilized Chicory and Oregano Decoction
Source: Plants (Basel). 2026 Feb 8;15(4):527. doi: 10.3390/plants15040527 (PMC12944019; doi:10.3390/plants15040527)
Supplement: Supplementary file 1 [file plants-15-00527-s001.zip › plants-4058266-supplementary.pdf]

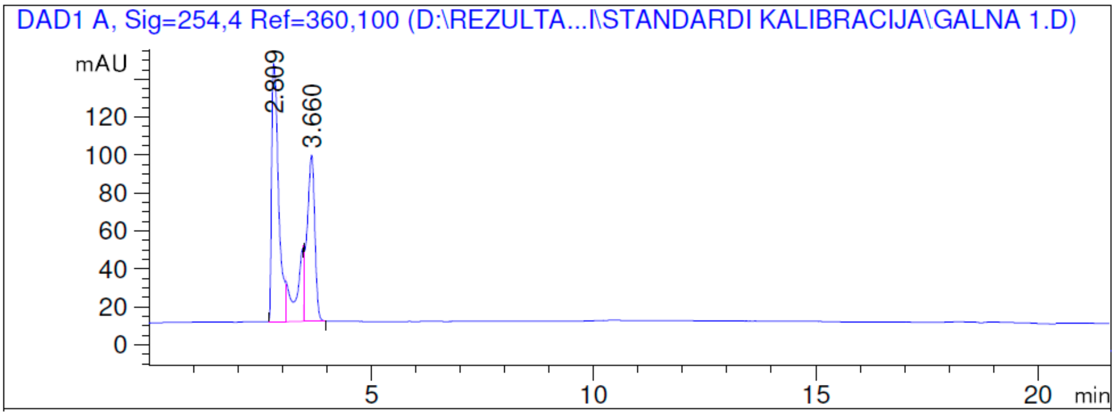

Figure S1. HPLC–DAD chromatogram of galic acid recorded at 254 nm.

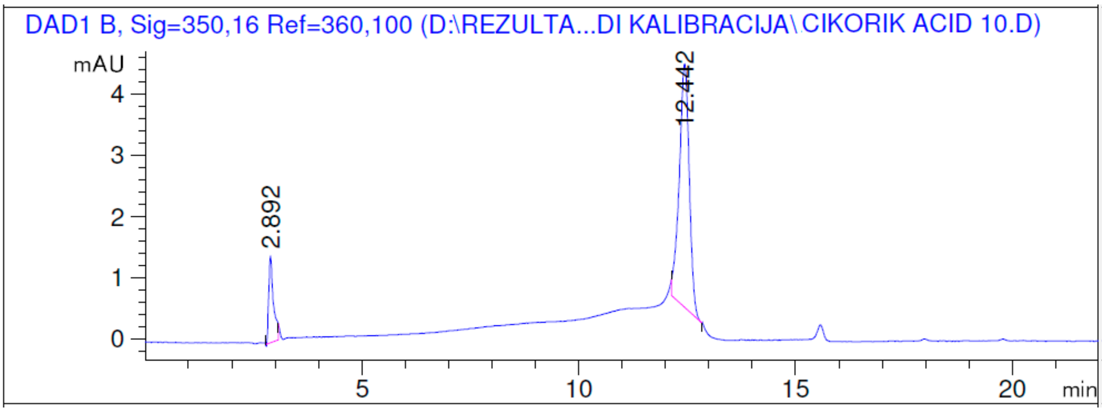

Figure S2. HPLC–DAD chromatogram of chicoric acid recorded at 350 nm.

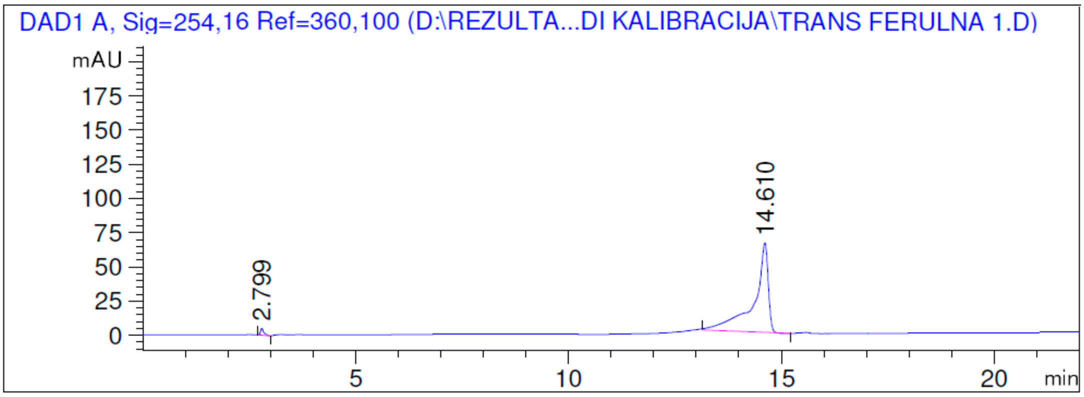

Figure S3. HPLC–DAD chromatogram of trans-ferulic acid recorded at 254 nm.

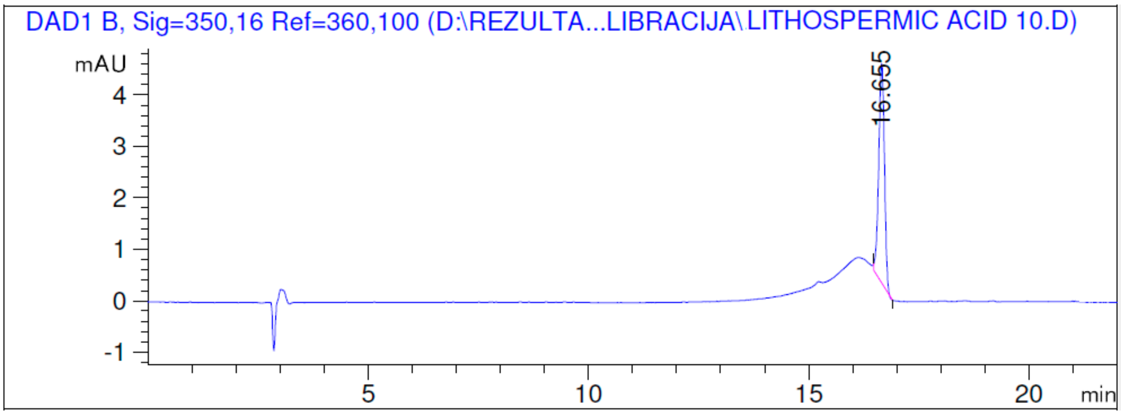

Figure S4. HPLC–DAD chromatogram of lithospermic acid recorded at 350 nm.

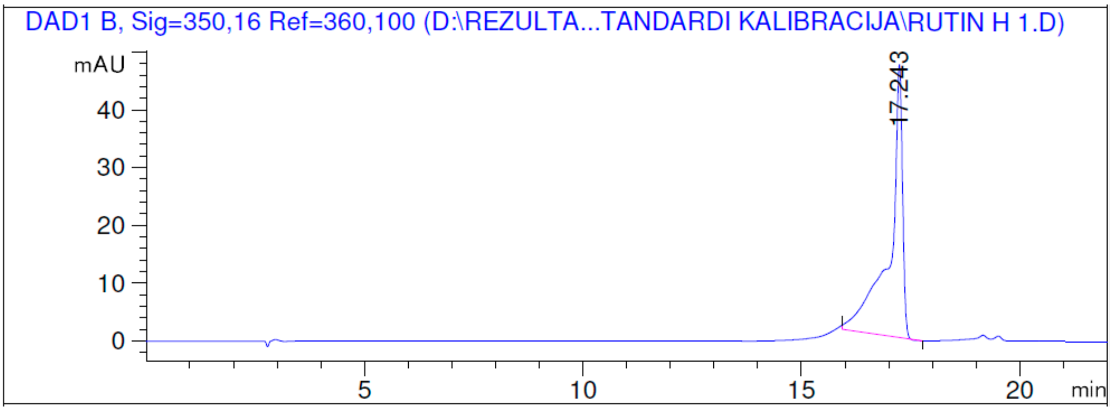

Figure S5. HPLC–DAD chromatogram of rutin recorded at 350 nm.

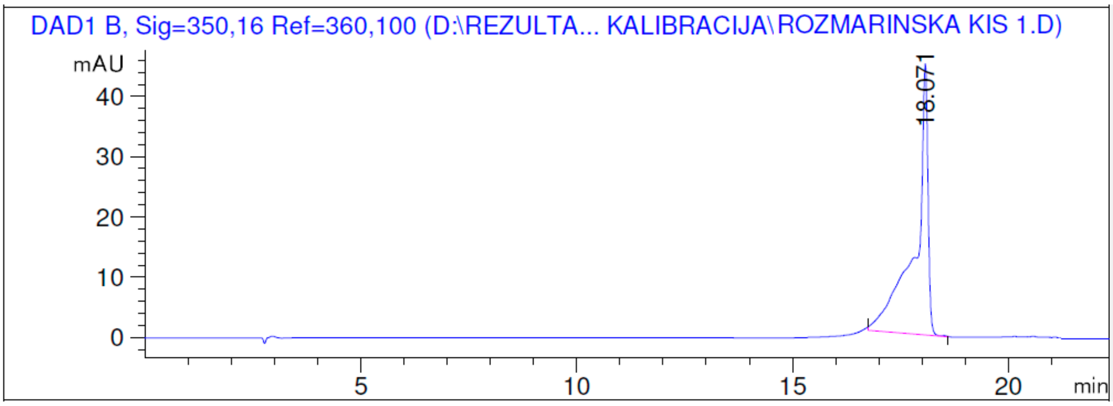

Figure S6. HPLC–DAD chromatogram of rosmarinic acid recorded at 350 nm.

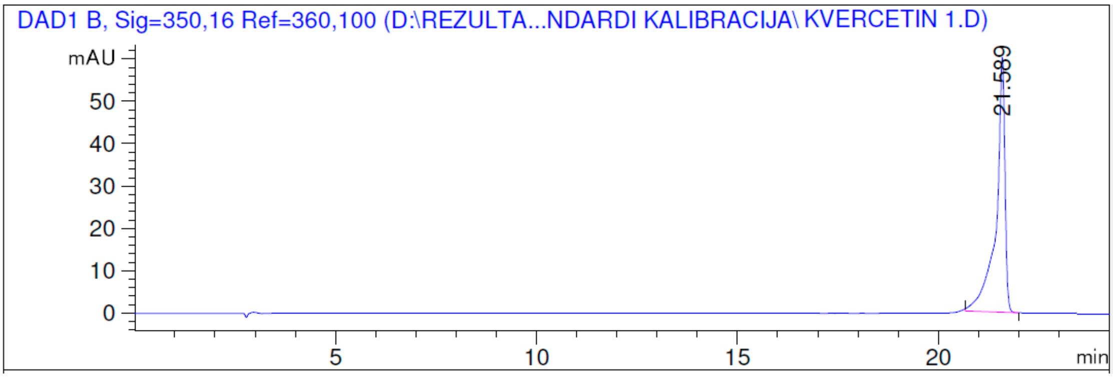

Figure S7. HPLC–DAD chromatogram of quercetin recorded at 350 nm.

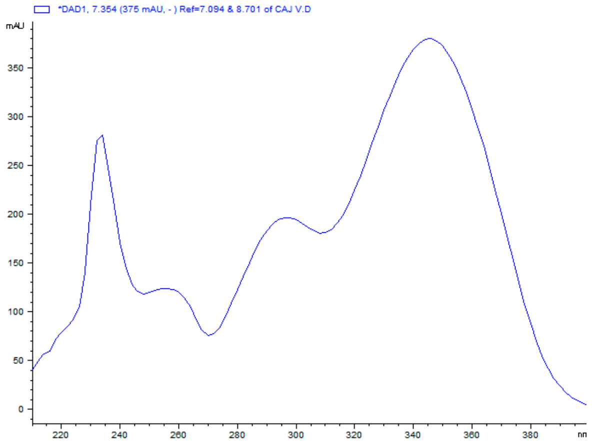

Figure S8. UV-Vis spectrum of caftaric acid derivative.

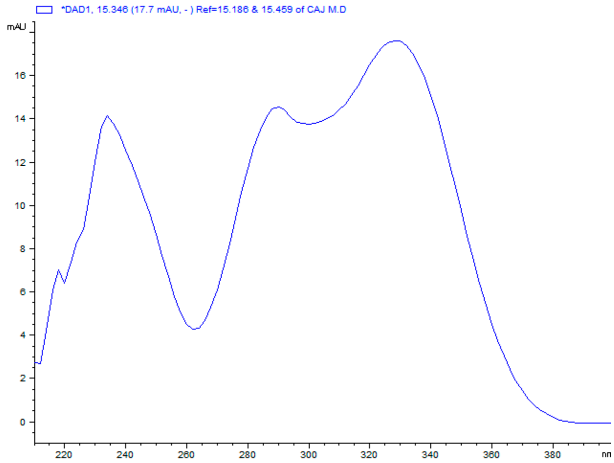

Figure S9. UV-Vis spectrum of chicoric acid derivative.

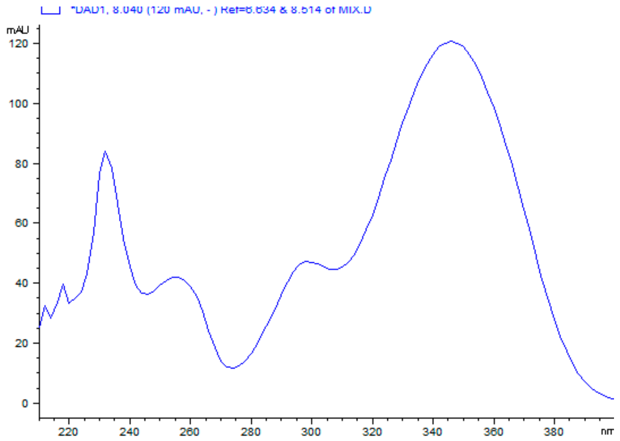

Figure S10. UV-Vis spectrum of caffeic acid derivative.

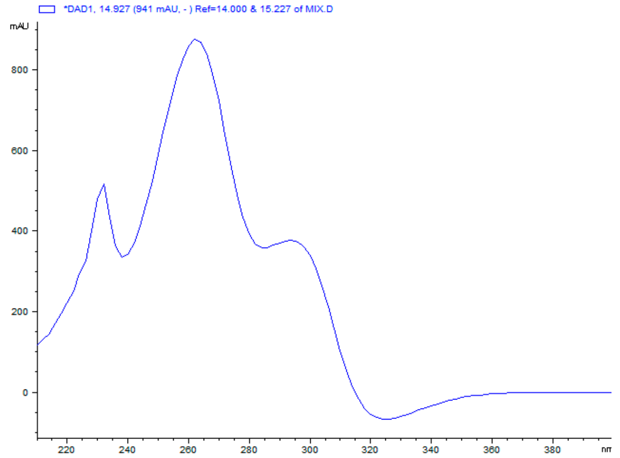

Figure S11. UV-Vis spectrum of ferulic acid derivative.

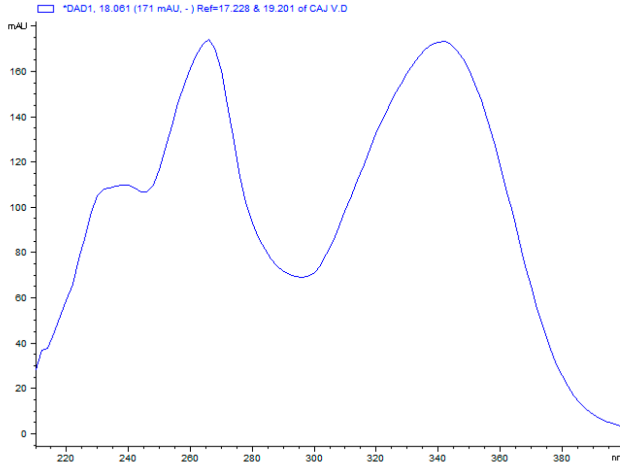

Figure S12. UV-Vis spectrum of apigenin-C-hexoside.

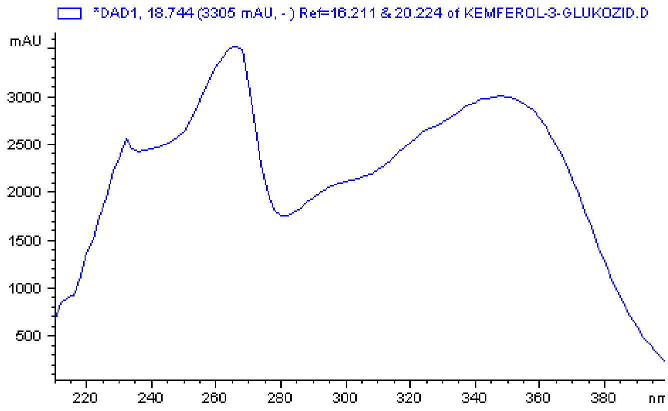

Figure S13. UV-Vis spectrum of kaempferol-3-O-glucoside.

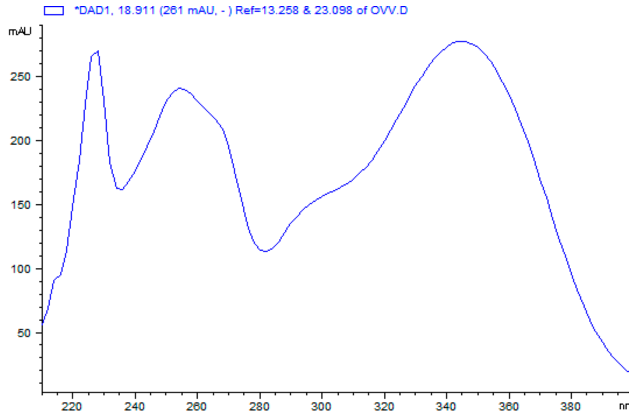

Figure S14. UV-Vis spectrum of kaempferol-glycoside derivative.

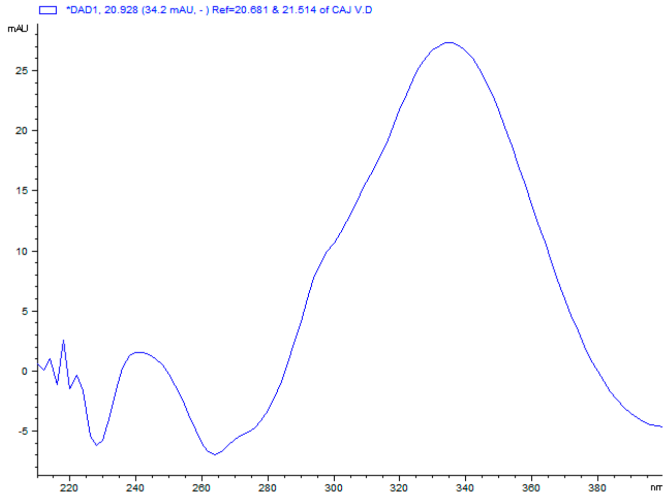

Figure S15. UV-Vis spectrum of quercetin derivatives.

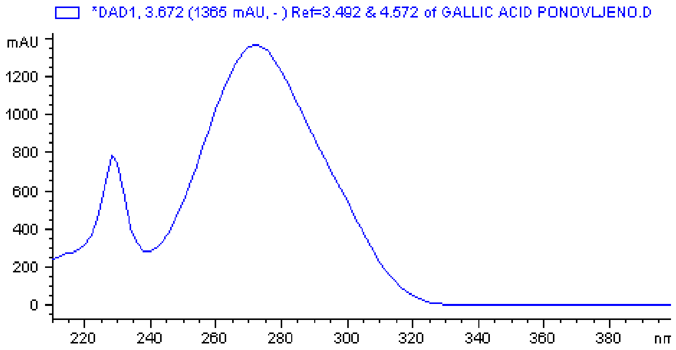

Figure S16. UV-Vis spectrum of gallic acid.

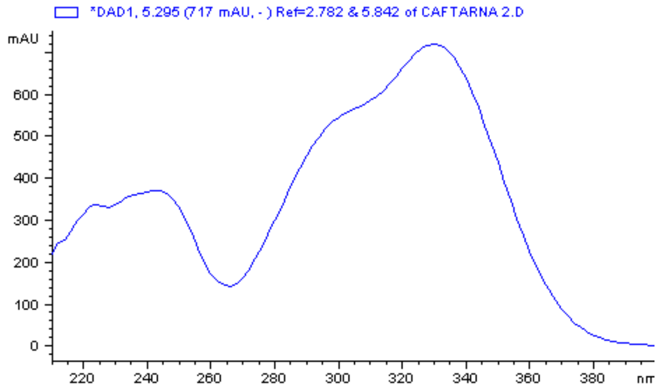

Figure S17. UV-Vis spectrum of caftaric acid.

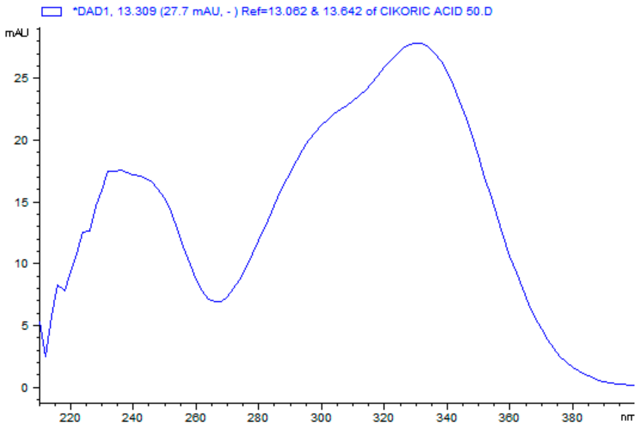

Figure S18. UV-Vis spectrum of chicoric acid.

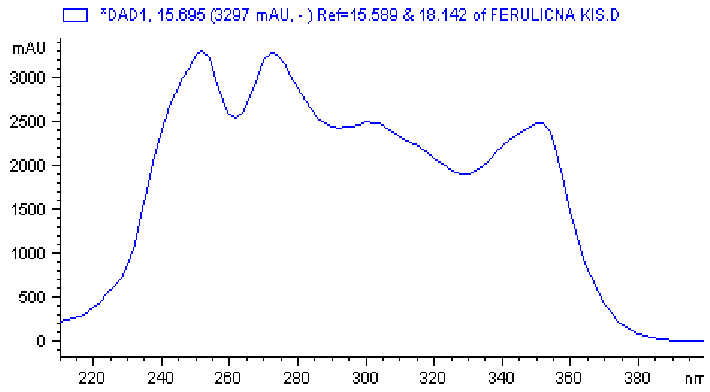

Figure S19. UV-Vis spectrum of ferulic acid.

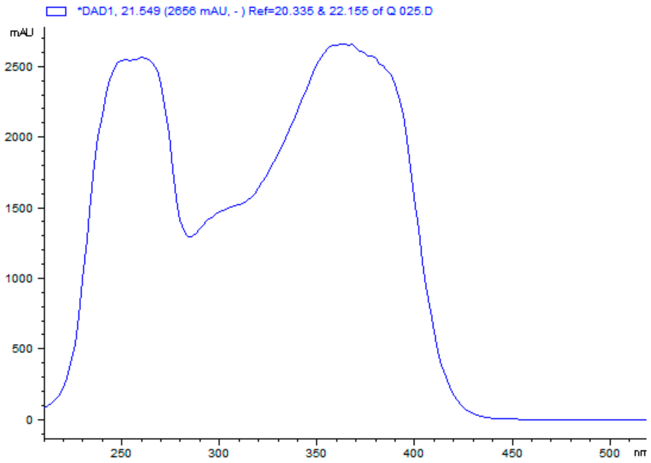

Figure S20. UV-Vis spectrum of quercetin.

Table S1. Regression equations, correlation coefficients, retention times and linear ranges for the reference standards used for HPLC quantification.

Retention Time (min)	Compound	Linear Regression Equations	Correlation Coefficients (R^2)	Linear range (µg/ml)
3.6	Gallic acid	y = 16.129x + 191.48	0,998	10 - 50
12.4	Chicoric acid	y = 4.4888x - 9.9619	0.9984	10 - 50
14.9	Trans ferulic acid	y = 30.11x - 91.704	0.9922	10 - 50
16.7	Lithospermic acid	y = 8.9365x - 30.024	0.9992	10 - 50
17.7	Rosmarinic acid	y = 19.923x - 130.29	0.999	10 - 50
18.9	Rutin	y = 20.292x - 125.65	0.999	10 - 50
22.4	Quercetin	y = 17.596x - 26.818	0.998	10 - 50
